# The importance of high‐quality heat adaptation research in females

**DOI:** 10.1113/EP093781

**Published:** 2026-04-21

**Authors:** Gabrielle E. W. Giersch, Toby Mündel

**Affiliations:** ^1^ US Army Research Institute of Environmental Medicine Natick Massachusetts USA; ^2^ Department of Kinesiology Brock University St. Catharines Ontario Canada

**Keywords:** heat acclimation, menstrual cycle, sex hormones

1

Heat acclimatization is the best tool available to reduce the risk of developing exertional heat illnesses and offset performance decrements during physical activity in the heat (Lorenzo et al., 2010; Minett et al., 2016; Periard et al., 2015). However, there is limited research investigating heat acclimatization in females, and of the limited investigations few have evaluated the potential influence of menstrual cycle phase or female sex hormones, which can influence thermoregulatory responses. It is unclear how female sex hormones influence adaptations garnered from heat acclimatization. There are various methods to induce heat acclimatization that have all been investigated extensively in men, including isothermic (controlled hyperthermia, whereby exercise intensity is altered to maintain core temperature within a certain range), clamped heart rate (whereby exercise is altered to maintain heart rate within a certain range), controlled work rate (whereby the exercise intensity and/or duration is fixed across heat acclimatization days/sessions) and passive (either hot air or hot water exposure for a given amount of time) modalities. It is unclear how various modalities influence adaptations or impact on performance during heat stress between sexes or within females.

In this issue of *Experimenal Physiology*, the *Myths and Methodologies* review by Mee and Flood (2026) highlights the importance of conducting high‐quality adaptation research in females, particularly accounting for sex hormone status. This review provides helpful recommendations for research, because the authors mention where specific mechanistic variables are being evaluated (e.g., the impact of female sex hormones on the magnitude or time course of adaptation) and where the strictest control is required. Ensuring proper classification of volunteers (i.e., naturally cycling, eumenorrhoeic, etc.), menstrual cycle phase (i.e., follicular or luteal) and sex hormone status is important to quantify the potential impact of these factors on the resulting adaptations accurately. This mechanistic understanding is vital to ensure that future practical research is appropriately designed, that sufficient evidence is used to ensure accurate recommendations for heat acclimatization in both sexes, and for a deeper understanding of the thermoregulatory impact of female sex hormones and/or the menstrual cycle.

Female sex hormones are well understood to influence thermoregulatory responses to exercise, with both menstrual cycle phase and contraceptive use influencing core temperature and skin blood flow responses, whereby elevated progesterone concentrations appear to increase the body temperature set point and elevated oestrogen concentrations increase cutaneous vasodilatation (Charkoudian & Stachenfeld, 2014). The practical effects of these hormonal fluctuations and associated thermoregulatory impacts are not consistent across stimuli and probably exhibit significant interindividual variability. However, these hormones might influence adaptations, because both core temperature and cutaneous blood flow responses are primary mechanisms of interest in heat acclimatization accounting for the enhanced heat dissipation mechanisms that prompt systematic decreases in core temperature. Another area that is under‐represented in research is the influence of long‐acting reversible contraceptives on thermoregulation, with very limited work evaluating their potential impact and the challenges associated with investigating these devices, which influence menstrual cycle frequency, length and symptomology.

An important factor outside the scope of the review mentioned above and the accompanying recommendations is the applied and more practical research, which also provides valuable insights into female physiology and potential sex differences in adaptation but is unable to quantify mechanistically the impact of sex hormone concentrations or menstrual phase on adaptations. In more generalizable research, it may not be possible to schedule heat acclimatization sessions in specific menstrual phases, with the appropriate level of tracking and control, because athletes and other physically active individuals/groups will probably not be able to adjust their training to a specific menstrual phase. This does not minimize the importance of those research questions or associated investigations; instead, it highlights their value in providing practical and generalizable data and recommendations.

Ultimately, more research is needed across the spectrum of required research control, methods of inducing heat acclimatization, potential impact of heat acclimatization on performance during exercise in the heat, accounting for menstrual cycle phase and female sex hormones, evaluating contraceptive use, using more practical and applied protocols without controlling for female sex hormones or menstrual cycle phase to elucidate fully the adaptations and factors that influence adaptations in females, in addition to males. This work by Mee and Flood (2026) provides important recommendations when control is necessary and highlights the importance of the topic and future research needed.

## AUTHOR CONTRIBUTIONS

Both authors approved the final version of the manuscript and agree to be accountable for all aspects of the work in ensuring that questions related to the accuracy or integrity of any part of the work are appropriately investigated and resolved. Both persons designated as authors qualify for authorship, and all those who qualify for authorship are listed.

## CONFLICT OF INTEREST

The author declares he has no conflicts of interest.

## FUNDING INFORMATION

No funding was received for this work.
